# Difference in the size of the placenta and umbilical cord between women with natural pregnancy and those with IVF pregnancy

**DOI:** 10.1007/s10815-017-1084-2

**Published:** 2017-11-14

**Authors:** Atsushi Yanaihara, Shota Hatakeyama, Shirei Ohgi, Kenichirou Motomura, Ryoma Taniguchi, Aguri Hirano, Shin Takenaka, Takumi Yanaihara

**Affiliations:** Yanaihara Women’s Clinic, 1-26-29 Ofuna, Kamakura, Kanagawa 247-0056 Japan

**Keywords:** IVF, Placenta size, Umbilical cord size, Velamentous insertion of cord

## Abstract

**Purpose:**

The purpose of this study was to compare the sizes of the placenta and umbilical cord in women with natural pregnancy versus those undergoing in vitro fertilization (IVF).

**Methods:**

Overall, 1610 cases of uncomplicated single pregnancies with vaginal delivery at ≥ 37 weeks of gestation were included in this study. The patients were divided into two groups: natural pregnancy group (*n* = 1453) and IVF pregnancy not including intracytoplasmic sperm injection (ICSI) treatment (*n* = 157). The groups were compared in terms of gestational week, maternal age, parity, maternal weight gain, prepregnancy maternal BMI, infant weight at birth, infant head circumference, placental weight, cross section of the placenta, cross section of the umbilical cord, insertion site of the umbilical cord, and umbilical cord length. Stepwise selection and multivariate logistic regression were used for statistical analysis to correct the result as an independent factor.

**Results:**

There was no difference in the size of the placenta and umbilical cord between women with natural pregnancy and with IVF, but the incidence of velamentous insertion of the cord was significantly increased in women with IVF pregnancy (adjusted odd ratio [AOR] 1.72, 95% confidence interval [CI] 1.08–2.72, *p* = 0.026).

**Conclusions:**

Although there is no difference in placental weight and cord size, velamentous insertion of the umbilical cord increases in IVF pregnancy and needs careful observation during the delivery process.

## Background

In Japan, one in 21 births results from in vitro fertilization (IVF) and the demand for IVF is increasing in various societies. According to the Japan Society of Obstetrics and Gynecology, infant weight is higher in case of IVF pregnancies, but the increase is not statistically significant. However, this has not been investigated in terms of its relationship to gestational week. IVF pregnancy often extends beyond the expected date of delivery, leading to increased medical intervention. It is reported that the weight of newborns born from IVF pregnancies is greater than that of babies born from routine pregnancies. Similarly, it is expected that the placenta in women with IVF pregnancies is larger than that in women with spontaneous pregnancies.

Haavaldsen et al. reported from 536,567 cases that the weight of the placenta was higher in cases of pregnancies resulting from assisted reproductive technology (ART) compared to those with spontaneous pregnancies, and the difference was independent of the gestational age at delivery [1]. Moreover, they suggested that the culture media for ART might have an effect on placental weight, and the conditions during very early embryonic life may influence birthweight and placental weight [2].

In this study, the sizes of the placenta and umbilical cord of women with spontaneous pregnancies and those with IVF pregnancies not including intracytoplasmic sperm injection (ICSI) treatment were compared in the same clinic and same environment.

## Material and method(s)

Overall, 1610 cases of uncomplicated single pregnancies with vaginal deliveries at ≥ 37 weeks of gestation between September 2015 and March 2017 were included in this study. The patients were divided into two groups: natural pregnancy group (*n* = 1453) and IVF pregnancy not including ICSI group (*n* = 157).

All IVF cases followed the same protocol and used the same culture medium. A mild stimulation protocol was performed using clomiphene and human menopausal gonadotropin (HMG; 150 units every other day [HMG150]; Ferring Pharmaceuticals, Tokyo, Japan) [3] for 3–4 times depending on the size of the follicle. Oocyte maturation was triggered using 1000 IU of human chorionic gonadotropin (hCG) (HCG 10000 U for injection; Fujipharma, Toyama-shi, Toyama). All embryos were frozen and transferred on day 5 (blastocyst stage) of the hormone replacement therapy cycle. Medicult Universal^®^ (Origio, Knardrupvej, Denmark) was used for the embryo culture. The hormone replacement was continued until 8 weeks of gestation. During the course of the pregnancy, none of the patients developed any complication.

The groups were compared with respect to several parameters such as maternal age, week of gestation, parity (primipara), maternal weight gain, prepregnancy maternal BMI, infant weight, infant head circumference, cross section of the placenta, placental weight, umbilical cord length, and cross section and insertion site of the umbilical cord.

We used multivariate logistic regression modeling to examine the association between pregnancy method (natural pregnancy or IVF pregnancy) and the factors mentioned above. First, multivariate logistic regression models were estimated using the method of conception as the dependent variable for all putative explanatory variables. If the correlation between some variables selected in the previous step was ≧ 0.8, the variables were excluded from further analysis. Thereafter, the model was developed using the stepwise selection method. For the final model, multicollinearity was assessed using the variance inflation factor. The model is presented with the odds ratio (OR), 95% confidence interval (CI), and *p* value for each predictor. We conducted all analyses using R version 3.3.2.1. A value of *p* < 0.05 was considered statistically significant in all tests.

This study was conducted with the approval of the Ethics Committee of Yanaihara Women’s Clinic and with patient consent (ERBY/4, 2014).

## Result(s)

Table 1 shows the comparison data of several factors between natural pregnancy and IVF pregnancy (mean ± SD).

**Table 1:** Comparison of the parameters for natural pregnancy and IVF pregnancy

Factor	Natural pregnancy (*n* = 1453)	IVF pregnancy (*n* = 157)
Maternal age (years)	35.2 ± 4.5	38.8 ± 3.8
Weeks of gestation	39.0 ± 1.2	39.1 ± 1.3
Primipara (*n*)	850	132
Maternal weight gain (kg)	9.2 ± 2.9	8.6 ± 2.7
Prepregnancy maternal BMI	20.7 ± 2.6	20.7 ± 2.4
Infant weight (g)	3016.2 ± 350.7	3037.9 ± 401.4
Infant head circumference (cm)	32.9 ± 1.4	33.0 ± 1.5
Cross section of the placenta (cm^2^)	340.0 ± 75.3	344.7 ± 85.7
Placental weight (g)	594.0 ± 103.2	603.5 ± 117.8
Umbilical cord length (cm)	55.4 ± 11.4	55.3 ± 12.8
Cross section of umbilical cord (cm^2^)	1.6 ± 1.0	1.6 ± 1.6
Velamentous insertion of cord, *n* (%)	145 (9.9)	32 (20.3)

The multivariate logistic regression showed significant difference in maternal age (adjusted odd ratio [AOR] 1.24, 95% confidence interval [CI] 1.18–1.29, *p* < 0.001), parity (AOR 0.21, 95% CI 0.13–0.33, *p* < 0.001), and insertion site of the umbilical cord (AOR 1.72, 95% CI 1.08–2.72, *p* = 0.026) (Table 2).

**Table 2:** Results of the multivariate logistic regression of the candidate factor

Factor	Adjusted OR (95% CI)	*p* value
Maternal age	1.24 (1.18–1.29)	< 0.001
Multipara	0.21 (0.13–0.33)	< 0.001
Velamentous insertion of cord	1.72 (1.08–2.72)	0.026

There were no differences in placental weight, infant weight, and umbilical cord length between women with natural pregnancies and those with IVF pregnancy.

## Conclusion

It has been reported that IVF/ICSI pregnancy can result in a high-risk delivery but the progression of pregnancy is normal [4–6]. IVF/ICSI pregnancy can increase the risk of postpartum hemorrhage [6, 7]. The purpose of this study was to understand the reasons for the high risks in IVF/ICSI deliveries. If placental and infant weights at birth are higher in IVF pregnancy, the medical intervention rate and postpartum hemorrhage might be increased.

In the advanced weeks of gestation, it is common for the infant weight and placental size to increase. With the increase in duration of gestation, the birthweight of infants resulting from ART pregnancy appears to be lower than that of infants resulting from natural pregnancy [1, 8].

Although Matsuda et al. did not mention the manner of conception, they reported the fetal/placental weight ratio in 53,650 Japanese pregnancies and found that inappropriately heavy placentas were found in female sex and in the nulliparity group, small-for-gestational age infants group, and infants from preeclamptic mothers group [9].

Factors like maternal age, racial difference, and sex of the infant can influence placental size [9, 10]. There are reports of placental size being linked to complications of pregnancy such as diabetes and pregnancy-induced hypertension [11, 12]. Maternal weight gain and prepregnancy maternal BMI may have a contributing influence on placental weight even in the absence of diabetes. In this study condition, maternal weight gain and prepregnancy maternal BMI appear to have no relationship with placental weight.

Eskild et al. suggested that the differences in placental weight between ART and natural pregnancy, independent of the gestation week at delivery, might be due to complications during the very early stage of pregnancy and not by conditions in the later part [2]. The microarray of tissue from the placenta showed that the expression of the six factors involved in the regulation of angiogenesis such as basic fibroblast growth factor and vascular endothelial growth factor receptor 3 was greater in the ART group than in the natural pregnancy group [13], which suggests that the expression of growth factors is strongly correlated to placental growth.

In velamentous umbilical cord insertion, the placental end of the cord consists of divergent umbilical vessels surrounded only by fetal membranes, with no Wharton’s jelly. Thus, it is assumed to increase the risk of severe complications, including fetal death, and it is seen in 3–5% of all deliveries. In our study, even in the normal pregnancy group, the rate of velamentous umbilical cord insertion was higher (9.9%) than that previously reported, but the reason is unclear. However, there have been many reports on increases in placental and cord abnormalities in IVF pregnancies [14–17], and our results are in line with these results (20.3%).

The pathogenesis of velamentous cord insertion is unknown. The most popular hypothesis is that the cord is initially inserted centrally, but its location progressively becomes peripheral as one half of the placenta actively proliferates toward the well-vascularized uterine fundus (trophotropism) while the other pole involutes; the umbilical cord is unable to follow the migration of the placenta.

The cause of increase in velamentous insertion of the cord in ART is also unknown; however, the manipulation involved in ART could be a reason. Several studies have reported a higher incidence of velamentous cord insertion in ART pregnancies, as the exact chronology of biological events required for proper blastocyst implantation is disturbed in more than one phase [15, 18, 19].

During delivery of IVF pregnancy, careful monitoring is necessary, and if the blood vessels are near the cervix, delivery via cesarean section might be a better option [20].

There was no difference in the placental weight and umbilical cord size; however, the rate of velamentous insertion of the cord increased significantly with IVF pregnancy. Further studies and improvement in IVF techniques may be necessary to decrease the incidence of the velamentous insertion of the cord.
